# Markers of neutrophil activation and extracellular traps formation are predictive of appendicitis in mice and humans: a pilot study

**DOI:** 10.1038/s41598-020-74370-9

**Published:** 2020-10-26

**Authors:** Michael Boettcher, Melina Esser, Julian Trah, Stefan Klohs, Nariman Mokhaberi, Julia Wenskus, Madgalena Trochimiuk, Birgit Appl, Konrad Reinshagen, Laia Pagerols Raluy, Michaela Klinke

**Affiliations:** Department of Pediatric Surgery, University Medical Center Hamburg-Eppendorf, UKE Medical School, Martinistrasse 52, 20246 Hamburg, Germany

**Keywords:** Biomarkers, Paediatric research, Gastrointestinal models

## Abstract

Appendicitis is one of the most frequent emergencies in pediatric surgery, yet current biomarkers for diagnosis are unspecific and have low predictive values. As neutrophils and extracellular traps (ETs) are an essential component of the immune defense against bacterial infections, and appendicitis is considered an inflammation reaction of the appendix, we hypothesized that neutrophil activation and NET formation play an essential role in appendicitis development and maintenance. Therefore, this pilot study aimed to establish a murine model of appendicitis and to evaluate ETs markers to diagnose appendicitis in mice and humans. The study used 20 (12 appendicitis- and 8 controls) 6-week old mice which underwent advanced appendicitis induction using a modified caecal ligation puncture procedure. During the study, cell-free DNA, neutrophil elastase (NE), myeloperoxidase (MPO), and citrullinated Histone H3 (H3cit) were assessed. Additionally, samples of 5 children with histologically confirmed appendicitis and 5 matched controls with catarrhal appendicitis, were examined for the same biomarkers. Moreover, NE, MPO, and H3cit were assessed histologically via immunofluorescence in mice and humans. All mice in the appendicitis group developed an advanced form of appendicitis with focal peritonitis. In mice and humans with appendicitis, markers of neutrophil activation and ETs formation (especially cfDNA, NE and H3cit) were significantly elevated in blood and tissue compared to controls. Ultimately, biomarkers correlated extremely well with tissue expression and thus disease severity. It appears that neutrophil activation and possibly NETs contribute to appendicitis development and biomarkers of neutrophil activation and ET formation reflect disease severity and thus could be used as biomarkers for appendicitis. However, large prospective clinical studies are needed to confirm our findings.

## Introduction

Acute appendicitis is the most common disease requiring emergency surgical treatment in children, as the lifetime risk of developing appendicitis is 7–8%, and the most common age for developing appendicitis is in the early teens^1^. Appendectomy is the treatment of choice. It requires general anesthesia and is associated with all known risks of abdominal operations. The complication rate of appendectomies (including wound infection, as well as abscess and adhesion formation) is around 20%^2,3^.

Despite the relatively high incidence of this emergency, appendicitis remains a complex diagnosis^4,5^. Especially in young children the disease may present atypically and many non-surgical conditions may mimic appendicitis^6^. In fear of potential perforation, surgeons tend to over-diagnose appendicitis, resulting in negative appendectomy rates between 10–25%^4,5^. Even though non-operative methods for appendicitis with antibiotics have been proposed, an early and reliable diagnosis of appendicitis is still essential, in light of global antibiotic resistance developments, in order to avoid unnecessary antibiotic treatment of children without appendicitis^7^.

In order to improve the diagnosis of appendicitis, various biomarkers have been evaluated. Typical laboratory tests are (1) white blood count (WBC), (2) polymorphonuclear leukocyte differential, and (3) C-reactive protein (CRP)^8^. These laboratory tests are known to be non-specific for appendicitis and only achieve high discriminatory power in combination with the patient’s clinical history, physical examination, and ultrasound^9^. However, recent studies have proposed alternative markers and methods such as Appy1, haptoglobin, interleukin-8, leucine rich alpha-2-glycoprotein, as well as metabolomic and cytokine profiling, but these are neither very predictive nor widely available^10–13^. Thus, biomarkers that are reliable and quickly processed are still needed.

During the first days of an infection like appendicitis, the body’s defense mechanism is almost entirely provided by the innate immune system, which mainly consists of one type of immune cell—the neutrophil. Neutrophils form the largest group of immune cells in the body and high numbers (around 10^11^) are produced daily^14^. In response to infection, neutrophils form extracellular traps (NETs), a tight net of nuclear material lined with cytotoxic proteins like myeloperoxidase (MPO) and neutrophil elastase (NE)^15,16^. This enables them to trap and destroy pathogenic organisms. However, NET formation also occurs inappropriately during sterile inflammation, i.e. after ischemia perfusion injuries, and can result in thrombosis, autoimmunity, and tissue damage^17–21^.

Recently, markers of NET formation have been shown to correlate with inflammatory disease like sepsis, osteomyelitis, or necrotizing enterocolitis^19,22,23^. Brinkmann et al. described NET in the appendix of adult patients with appendicitis^16^. As the role of neutrophil activation and ETs formation in pediatric appendicitis has yet to be investigated, the aim of this study was to determine whether biomarkers of neutrophil activation and ETs formation could be utilized to diagnose appendicitis. Therefore, both (1) an appendicitis mouse model was established and (2) pediatric human appendicitis samples were evaluated.

## Methods

### Study design

The study was approved by the ethics committee Hamburg State Administration for animal research (100/17). A total of 20 mice were utilized for the experimental model and were held within the animal facility, according to environmental parameters established and dictated by the German guide for the care and use of laboratory animals (Tierschutzgesetz)*.*

Additionally, histological examinations of 10 children that underwent appendectomy at the Department of Pediatric Surgery of the University Medical Center Hamburg-Eppendorf in 2018 were included. Of these, five patients were intraoperatively diagnosed with perforated appendicitis with localized free fluid (Grad 3 according to disease severity score). Functioning as a matched control, five additional patients were diagnosed intraoperatively with catarrhal appendicitis (Grad 0 according to disease severity score) and used as controls^24^. Matching was performed based on age, gender, and duration of symptoms. Patients with comorbidities especially immunosuppression were excluded from this study. Ethics approval (PV5891) had been obtained from the medical research ethics committee of Hamburg (Ethik-Kommission der Ärztekammer Hamburg). All examinations in humans were in accordance with their guidelines and with the 1964 Helsinki declaration and its later amendments. Witten informed consent was obtained from the parents or legal guardians.

### Animal procedures

**Figure 1:**
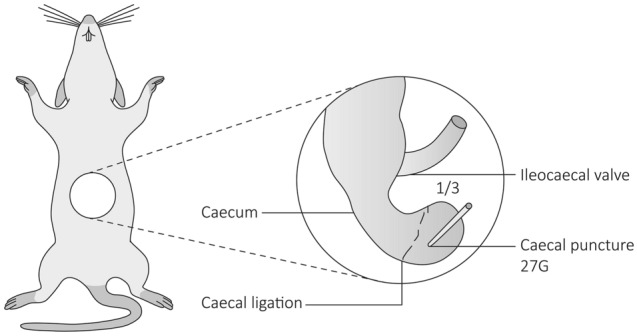
Experimental design to induce advanced appendicitis. After median laparotomy, the caecum identified. The caecum was ligated at the last third which includes the caecal patch (the murine equivalent of human appendix) and punctured with a 27G needle. In controls, a median laparotomy was performed without caecal ligation and puncture. All animals were euthanized on day four.

The study made use of 6-week old C57BL/6 J mice, which were housed within the animal care facility with food and water ad lib. In order to induce advanced appendicitis a modified caecal ligation puncture (CLP) procedure was performed as shown in Fig. 1^25^. To assess the development of appendicitis and NET formation, blood samples were taken every 24 h in alternating mice. All animals were euthanized after sedation with isoflurane gas (Forene 100%, AbbVie) on day four after CLP. In order to control for other factors that may influence inflammation and NET formation, a control group was included that were subjected to median laparotomy.

#### Sample collection and storage

##### Blood

For the time course of the biomarkers, blood sampling was performed via retroorbital venous sinus. Upon euthanasia, blood samples were collected in test tubes containing ethylenediaminetetraacetic acid (EDTA) through decapitation and processed immediately. The samples were centrifuged at 2000 relative centrifugal force for 10 min at room temperature in order to separate plasma. It was preserved at a temperature of − 80 °C until further analysis. Blood plasma was used to assess levels of cell free DNA (cfDNA), neutrophil elastase (NE), myeloperoxidase (MPO), and citrullinated Histone 3 (H3cit) via ELISA. NE and MPO are markers of neutrophil activation, while cfDNA and H3cit are surrogate markers of NET formation. Plasma was collected and analyzed for cfDNA based on a previously described method^26^. Additionally, the WBC and CRP was assessed for each subject.

##### Intestinal tissue

After blood collection, the animals were dissected using a midline incision and the bowel was prepped with aid of a light microscope to evaluate the intestine for signs of appendicitis. At this time, the morphologic analysis was performed and captured using a 4 K/12-megapixel camera. The ligated coecum was then evenly distributed into test tubes containing: (1) Bouin and (2) liquid nitrogen gas. The shock frozen samples (liquid nitrogen gas) were immediately stored at − 80 °C until further processing.

#### Morphologic analysis of the intestine

Scoring of macroscopic appendicitis manifestation occurred during dissection and was performed by two observers blinded to the subject’s test group. As all animals showed a gangrenous appendix with focal abscess formation, no further differentiation was performed.

#### Tissue preparation and evaluation

##### Histological analysis

Upon tissue fixation in Bouin’s solution, intestinal tissue samples were dehydrated overnight and embedded in paraffin. The intestinal tissue was then cut into 3 µm thick sections and applied to slides for further analysis.

##### Immunofluorescence staining (NE, MPO, H3cit)

3 μm-paraffin tissue sections were prepared for immunfluorescence staining by deparaffinization and rehydration. Antigen retrieval was assessed by incubating murine specimen with Target Retrieval Solution pH6 (Dako, Santa Clara, USA) for 90 min at 60 °C, following a cooling step of about 30 min. After rinsing sections 2 × 3 min with TBST, blocking of the probes was performed with Protein Block Solution (BioGenex, Fremont, USA) for 30 min at room temperature (RT). Tissue specimen were further incubated with either Isotype- or antigen-specific-antibodies at 4 °C (all Abcam, UK): rabbit anti-mouse NE antibody was used at a dilution of 1:200, whereas mouse anti-mouse MPO- and rabbit anti-mouse H3cit- antibodies were diluted 1:50. 18 h later, sections were rinsed 3 × 3 min with TBST and incubated after application of 1:200 AlexaFluor647-coupled secondary antibody at RT for 30 min (Abcam, UK). After a 3 × 5 min rinsing-step with TBST, nuclei were counterstained by incubating probes with DAPI for 5 min at RT. Finally, slides were rinsed 1 × 5 min with PBS and 1 × 5 min with H_2_O and mounted with Fluoromount-G (Southern Biotech, Birmingham, USA). Images of fluorescently labeled sections were acquired with an inverted fluorescence microscope (Axiovert 200 M, Objective: LD Plan-Neofluar 20x/0.4, Zeiss). Digitized MPO, NE, and H3cit slides were then evaluated in a semi-quantitative and blind fashion in four different areas of the affected intestine as compared to shams established previously^19^:

- None (0)—no signs of tissue staining
- Mild (1)—small amount of tissue staining
- Moderate (2)—medium amount of tissue staining
- Severe (3)—large amount of tissue staining

##### Human samples

Blood samples of patients were taken after informed consent was obtained at the time of the first presentation. The blood was analyzed for cfDNA, MPO, NE, and H3cit using ELISA. After optimization only very small volumes of EDTA plasma are needed: cfDNA 10 ul, NE 2 ul, MPO 2 ul and H3cit 10 ul. Intestinal samples were collected during surgery upon removal of the appendix. The intestinal samples were stained according to the previous description of mice sample preparation (HE, NE, MPO, H3Cit) and analyzed by a pathologist blinded to the study.

### Statistics

All data were analyzed using SPSS Statistics 26 (IBM, NY, USA) and GraphPad Prism 8 (GraphPad, CA, USA). As this is a pilot study, no formal power calculation was performed. Differences between groups were calculated using t-test and ANOVA with Dunnett’s post-hoc. Results are presented as mean ± standard deviation (SD). For association between factors, Spearman's Rho was utilized. Survival analysis was calculated using the Mantel-Cox test. Moreover, propensity score matching was performed using the nearest neighbor with caliper of 0.4 for age, gender, and duration of symptoms. The level of significance was set at < 0.05.

## Results

In total 20 mice were utilized (12 appendicitis - 8 control group). In the control group most animals (7/8) and in the appendicitis half (6/12) survived the procedure until day four (*p* = 0.088). All mice in the appendicitis group developed an advanced form of appendicitis with focal peritonitis. No control animals showed any signs abdominal infection.

There were no significant differences between both groups of human subjects, namely (1) perforated appendicitis with localized free fluid and (2) catarrhal appendicitis (age (controls 13.40 (4.04) vs. appendicitis 13.40 (4.51) years, *p* = 1.00), gender (controls 3/5 vs. appendicitis 3/5 female, *p* = 1.00), duration of symptoms (controls 28.80 (20.08) vs. appendicitis 31.20 (16.10) hours, *p* = 0.84)). However, a significant difference was found for CRP (controls 13.60 (16.83) vs. appendicitis 60.20 (33.23) mg/dl, *p* = 0.023), but not for white blood count (controls 9.22 (5.86) vs. appendicitis 13.04 (3.00) × 10^9^/l, *p* = 0.23).

**Figure 2:**
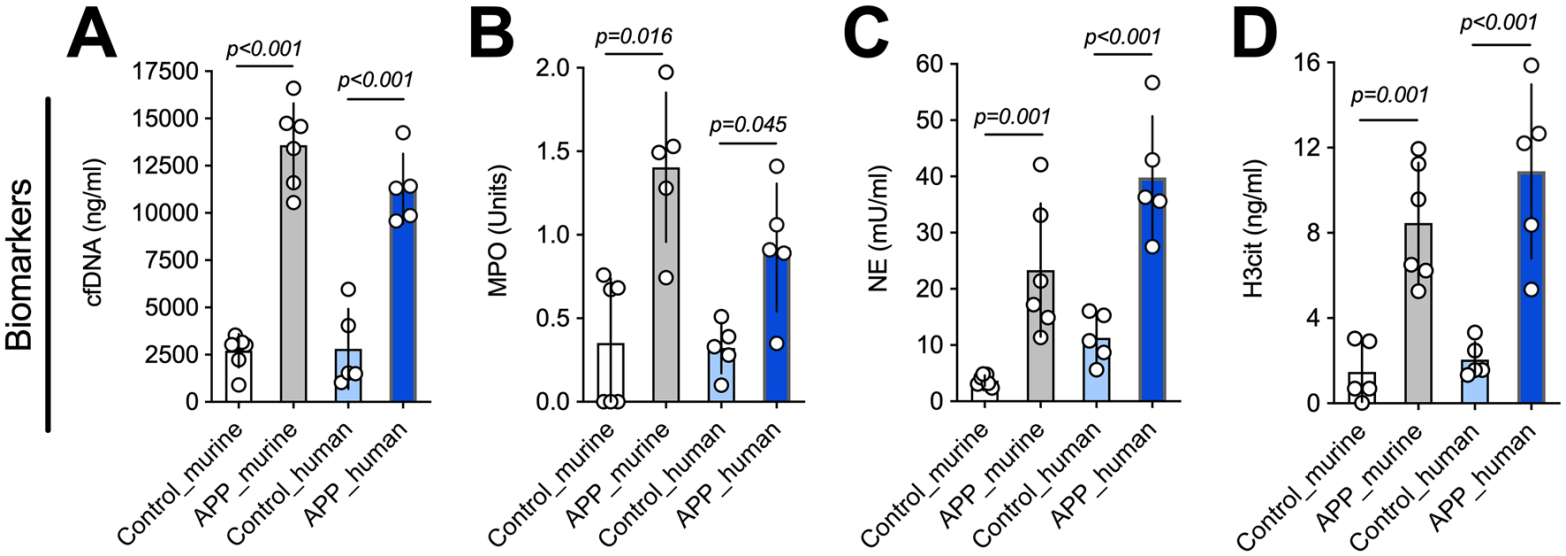
Markers of neutrophil activation and extracellular trap formation are significantly elevated in mice and humans with appendicitis. (**A**) CfDNA, a surrogate marker of NET formation, was significantly elevated in mice and humans with appendicitis. (**B**, **C**) MPO and NE are markers of neutrophil activation. Both markers were significantly elevated in mice and humans. (**D**) H3cit is the most specific marker of NET formation, and was significantly elevated in mice and humans with appendicitis compared to controls. Data shown as Mean ± SD. Statistics: t-test.

In mice and humans, biomarkers of NET formation correlated significantly with appendicitis. More specifically, cfDNA (Fig. 2A), MPO (Fig. 2B), NE (Fig. 2C), and H3cit (Fig. 2D) were significantly elevated in mice and humans with appendicitis compared to controls. The time course of CfDNA and MPO in mice is shown on supplement 1. CfDNA appears to peak early after the CLP procedure, indicating a possible bias as a result of the laparotomy (supplement 1A). MPO, however, increased with time showing significant differences at day 4 after CLP (supplement 1B).

**Figure 3:**
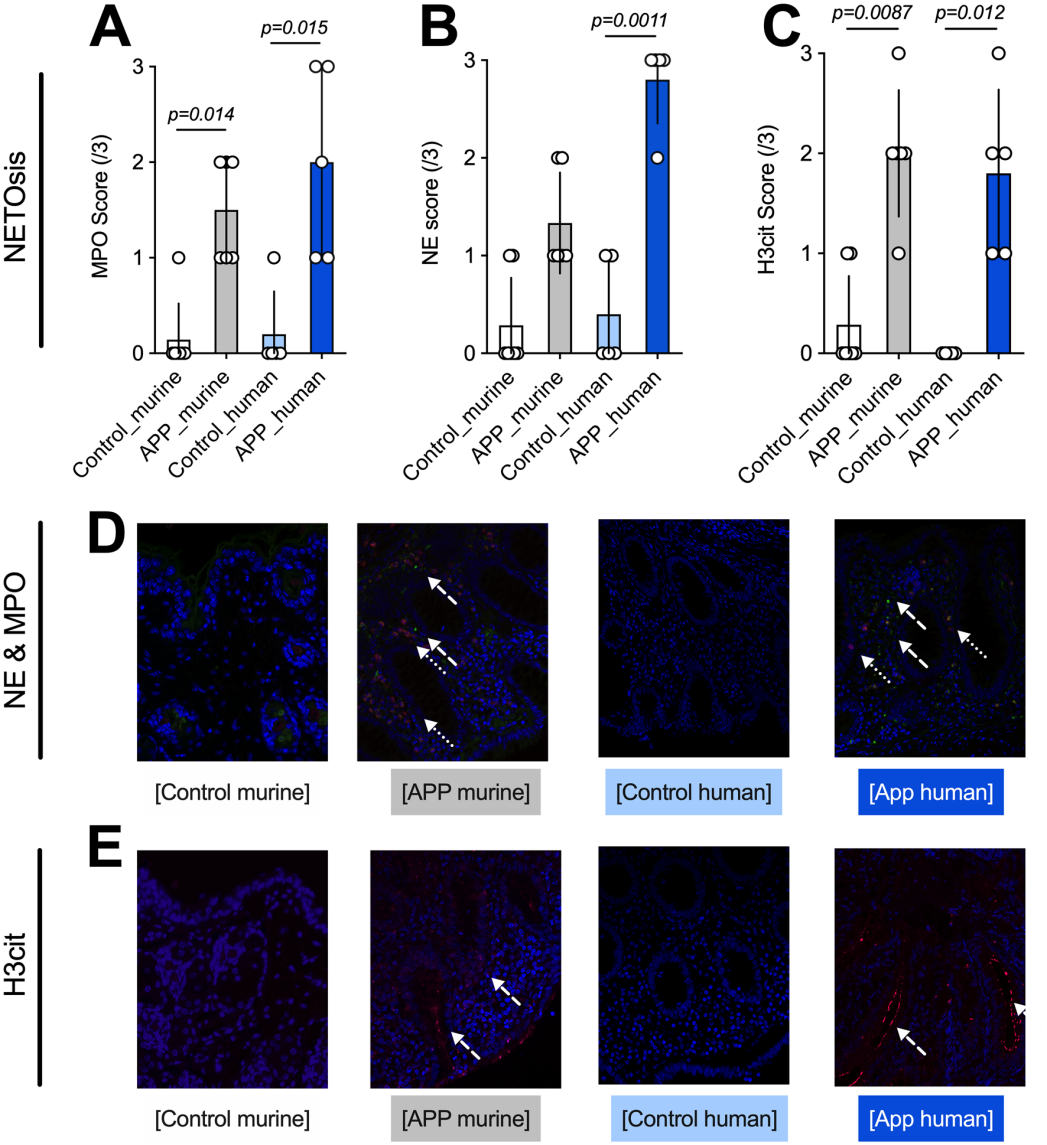
Histology of mice and humans showing a significant elevation of neutrophil activation and NET markers in mice and humans with appendicitis compared to controls. (**A**, **B**, **D**) Markers of neutrophil activation (MPO, green, NE, red) were significantly elevated; especially in human subjects with appendicitis compared to controls. (**C**, **E**) H3cit (red) scores were significantly higher in mice and humans with appendicitis than in scontrols. Data shown as Mean ± SD. Statistics: Mann–Whitney test.

Moreover, mice and humans had similar histopathological findings. Components of NET formation, measured using expression levels of MPO (Fig. 3A,D), NE (Fig. 3B,D), and H3cit (Fig. 3C,E) were increased in both mice, and especially humans, with appendicitis, and were significantly elevated when compared to controls as shown in Fig. 3.

Ultimately, biomarkers and histological markers of NET formation showed an excellent correlation in mice MPO (r = 0.77, *p* = 0.0052), NE (r = 0.85, *p* = 0.0002), H3cit (r = 0.89, *p* = 0.0002), and in humans MPO (r = 0.91, *p* = 0.0003), NE (r = 0.79, *p* = 0.0064), H3cit (r = 0.76, *p* = 0.01).

## Discussion

The current pilot study shows that markers of neutrophil activation and ETs formation are significantly increased in humans and mice with appendicitis compared to controls. This is particularly the case for markers of cfDNA, NE, and H3cit, which were significantly elevated in both humans and mice. Additionally, the above-mentioned markers were highly expressed in intestinal tissue of subjects and mice with appendicitis. Ultimately, neutrophil activation and ETs formation markers in blood correlated extremely well with tissue expression, and thus disease severity.

The current study suggests that markers of neutrophil activation and extracellular trap formation like cfDNA, NE, MPO, and H3cit may be excellent predictors of appendicitis. For the determination of these markers only very small blood volumes are needed (less than 500ul). Cell free DNA appears to be particularly interesting. It is comprised of short-lived fragments of DNA produced secondary to cellular necrosis and apoptosis as well as NET formation^21,27–30^. Healthy people show minimal levels due to the clearance of apoptotic cells by phagocytes^31^. In the setting of infection however, rapid cellular turnover, increased apoptosis and impaired clearance of dying cells leads to an accumulation of cfDNA^32^. Cell-free DNA has been shown to be an excellent early and prognostic marker of sepsis, and been associated with adverse outcome of pneumonia, osteomyelitis, and burn and trauma patients^21,33–38^. Lastly, cfDNA has recently been linked to intestinal infection in mice and humans^19,23,39^.

Moreover, markers of neutrophil activation and NET formation like NE, MPO and H3cit appear to be closely linked with intestinal inflammation and could possibly be more predictive than current markers like CRP or WBC. They have been evaluated in neonates with necrotizing enterocolitis and showed a significant correlation with intestinal inflammation and disease severity^19^. Moreover, NETs have been directly linked with inflammatory bowel disease (IBD) in adult and pediatric patients^40,41^. However, they have not been evaluated as systemic biomarkers in these patients. As neutrophils and NETs are directly affected by intestinal inflammation, one might assume that the neutrophil-associated biomarker might be superior to current markers like CRP or WBC in predicting and differentiating appendicitis. However, future studies should evaluate if NETs cause, drive or are merely a byproduct of appendicitis.

In the current study, human subjects with appendicitis demonstrated significantly higher CRP levels, but no difference in WBC than their matched controls. Most previous studies have identified that a high WBC and granulocyte count, as well as increased CRP concentration, predict complicated appendicitis^9,42^. In fact, in a meta-analysis evaluating the diagnostic value of serum markers for more than 1000 patients with perforated or non-perforated appendicitis, CRP was found to have the best discriminative capability in diagnosing appendicitis, followed by WBC and procalcitonin, which is reflected by this pilot study^43^. However, it remains very difficult to differentiate simple from complicated appendicitis; even when using CT imaging and considering its associated risk^44^. Neutrophil activation and NETs marker could close this diagnostic gap and, therefore, should be evaluated in future for this measure.

The main limitation of this pilot study is the sample size of human subjects. Naturally, a much larger cohort will be needed to confirm our results. Moreover, the modified CLP procedure used in our study might be a good model in order to induce advanced appendicitis, however, the model is not a perfect model of appendicitis as it only induces advanced and not simple appendicitis with typical disease progression. Thus, our study’s findings are not applicable to the patient population with simple appendicitis, even though histopathology showed similar findings in mice after the modified CLP procedure was applied as in humans with confirmed appendicitis. Moreover, both mice and humans demonstrated similar levels of neutrophil activation and extracellular trap formation in their blood samples. Nonetheless, in order to assess the involvement of NETs in simple appendicitis the murine appendicitis induction models by Watson et al. or Cheluvappa et al., which include identification and isolated ligation of the caecal patch without puncture, may be better suited^45,46^. Furthermore, future studies should further evaluate the role of neutrophil activation and NETs in appendicitis pathology using genetic knockout models.

In conclusion, our results indicate that markers of neutrophil activation and extracellular trap formation are excellent biomarker of appendicitis, as cfDNA, NE, and H3cit correlated perfectly with clinical and histological diagnosis. In particular, the readily accessible cfDNA seems to have a very promising future in appendicitis diagnostics, so that future studies with much larger sample sizes are needed to confirm our findings.

## Supplementary information

Supplementary Information.
