# Supplementary material for: Markers of neutrophil activation and extracellular traps formation are predictive of appendicitis in mice and humans: a pilot study
Source: Sci Rep. 2020 Oct 26;10:18240. doi: 10.1038/s41598-020-74370-9 (PMC7588418; doi:10.1038/s41598-020-74370-9)
Supplement: Supplementary file 1 — Supplementary Information. [file 41598_2020_74370_MOESM1_ESM.docx]

**Markers of neutrophil activation and extracellular traps formation are predictive of appendicitis in mice and humans: a pilot study**

Michael Boettcher M.D. Ph.D.^1*^, Melina Esser^1*^, Julian Trah M.D.^1^, Stefan Klohs M.D.^1^, Nariman Mokhaberi M.D.^1^, Julia Wenskus^1^ M.D., Madgalena Trochimiuk^1^, Birgit Appl^1^, Konrad Reinshagen M.D. Ph.D.^1^, Laia Pagerols-Raluy Ph.D.^1*^, Michaela Klinke M.D.^1*^

**
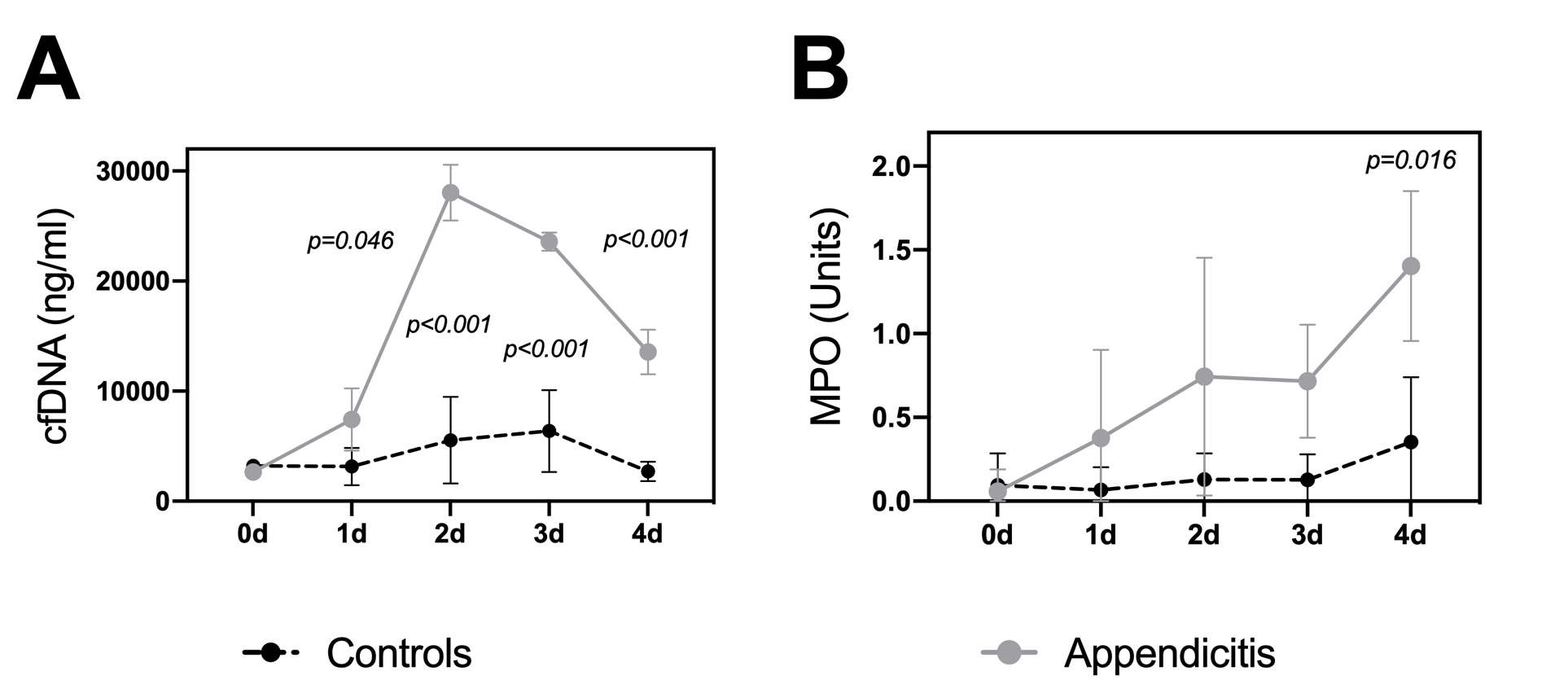
**

Supplement 1: **Time course of cfDNA and MPO in mice.** CfDNA showed a peak two days after CLP procedure. Whereas MPO increased over time. Data shown as Mean±SD. Statistics: t-test.
